# Identifying ultrasound and clinical features of breast cancer molecular subtypes by ensemble decision

**DOI:** 10.1038/srep11085

**Published:** 2015-06-05

**Authors:** Lei Zhang, Jing Li, Yun Xiao, Hao Cui, Guoqing Du, Ying Wang, Ziyao Li, Tong Wu, Xia Li, Jiawei Tian

**Affiliations:** 1 Department of Ultrasound, The Second Affiliated Hospital of Harbin Medical University, Harbin, Heilongjiang, China; 2 Department of Ultrasonic medicine, The 1st Affiliated Hospital of Heilongjiang University of Chinese Medicine, Harbin, Heilongjiang, China; 3 College of Bioinformatics Science and Technology, Harbin Medical University, Harbin, Heilongjiang, China; 4 Department of general surgery, The Second Hospital of Hebei Medical Universtiy, Shijiazhuang, Hebei, China

## Abstract

Breast cancer is molecularly heterogeneous and categorized into four molecular subtypes: Luminal-A, Luminal-B, HER2-amplified and Triple-negative. In this study, we aimed to apply an ensemble decision approach to identify the ultrasound and clinical features related to the molecular subtypes. We collected ultrasound and clinical features from 1,000 breast cancer patients and performed immunohistochemistry on these samples. We used the ensemble decision approach to select unique features and to construct decision models. The decision model for Luminal-A subtype was constructed based on the presence of an echogenic halo and post-acoustic shadowing or indifference. The decision model for Luminal-B subtype was constructed based on the absence of an echogenic halo and vascularity. The decision model for HER2-amplified subtype was constructed based on the presence of post-acoustic enhancement, calcification, vascularity and advanced age. The model for Triple-negative subtype followed two rules. One was based on irregular shape, lobulate margin contour, the absence of calcification and hypovascularity, whereas the other was based on oval shape, hypovascularity and micro-lobulate margin contour. The accuracies of the models were 83.8%, 77.4%, 87.9% and 92.7%, respectively. We identified specific features of each molecular subtype and expanded the scope of ultrasound for making diagnoses using these decision models.

Breast cancer is one of the major causes of death for females worldwide and its incidence has been increasing^1^. This disease follows a diverse natural history and is variably responsive to treatments^2^. The limitations of traditional histological classification have led to the development of a new molecular classification, which has demonstrated the existence of four main subtypes^2^: Luminal-A type (LA), Luminal-B type (LB), Epidermal growth factor receptor 2-amplified type (HER2), and Triple-Negative type (TN). Gallen *et al.*^3^ found that the LA tumors with high Ki-67 expression (Ki-67 ≥ 14%) should be classified as LB subtype. Clinically, LA subtype is the most common, and several genomic tests exist for assisting in predicting patient outcomes upon receiving endocrine therapy. LB patients can benefit from neoadjuvant chemotherapy. HER2 patients generally show excellent clinical outcomes when given an effective therapeutic, trastuzumab, which targets the HER2 gene. TN subtype is a group that only has chemotherapy options^4^,^5^,^6^.

Although pathological diagnosis is the “gold standard” for distinguishing the molecular subtypes of breast cancer, it is invasive and might cause physical and psychological discomfort in patients. Accordingly, the development of a non-invasive method will significantly improve the diagnostic procedure. The utility of ultrasound method for the diagnosis of breast lesions has increased over the past decade^7^. Ultrasound, with its high level of safety and low cost, is becoming the preferred method for both physicians and patients. Reports have indicated that improvements in ultrasound technologies might make it possible to highly sensitively differentiate malignant solid breast masses from benign ones based on their different ultrasound features^8^. A few studies have examined the correlation between ultrasound features and certain types of biological behavior. Irshad *et al.* found that posterior shadowing is strongly associated with Estrogen Receptor-positive (ER+) and low-grade tumours, whereas posterior enhancement is strongly associated with high-grade tumours and a moderate risk of being receptor negativity^9^. Wang *et al.* demonstrated that in contrast to ER-negative HER2-negative tumours, ER-negative HER2-positive tumours were more likely to have spiculated margins with calcification and a higher cancer stages^5^. Ko *et al.* suggested that TN breast cancers have more circumscribed margins, are hypoechoic, and exhibit less calcification and posterior shadowing^10^. The above studies suggested the possibility of determining the relationship between patient characteristics and the individual molecular situation, molecular ordination or TNBC imaging features. However, although the ultrasound features of breast cancer might correlate with the molecular subtypes identified by immunochemistry (IHC) examination, the characterization of the four breast cancer molecular subtypes by ultrasound imaging and clinical modality might be complex^11^. As such, identifying the subtypes might require the assessment of a combination of characteristics, similar to those used for differentiating benign and malignant tumours.

To accurately detect the different features of breast cancer molecular subtypes, efficient statistical methods and computational algorithms for analysing the massive amount of clinical data available need to be developed. Decision trees are one of the most popular classification techniques for multiple features in data mining and machine learning^12^, and these can be converted to rule sets to improve interpretation. However, existing attempts to apply decision trees to classification using gene expression data have shown that single-tree algorithms are not sufficient for high accuracy and stability. In this study, we propose the ensemble decision approach that integrated multiple decision trees based on an ensemble decision theory to select the special features of each subtype^13^. We obtained multiple feature sets from the training sets by a resampling technique, and integrated the multiple feature sets to produce a combination of features of each subtype by the ensemble decision approach. We not only constructed the models but also obtained high accuracy with the models, and considered that the ensemble decision approach might have significant utility for ultrasound diagnosis of breast cancer in the future.

## Results

### The general description and feature distribution of the four subtypes

**Figure 1:**
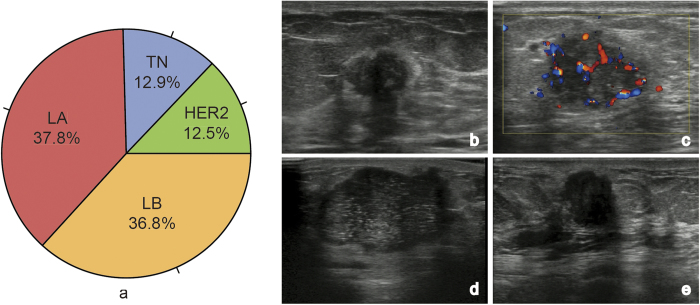
a : A pie chart of the four breast cancer subtypes. **b**–**e** are the ultrasound pictures of the LA, LB, HER2 and TN subtypes, respectively. **b** An LA patient showing the halo and post-acoustic shadowing. **c** An LB patient with vascularity. **d** An HER2 patient with calcification and enhanced post-acoustic. **e** A TN patient showing the micro-lobulate margin and oval shape.

**Figure 2:**
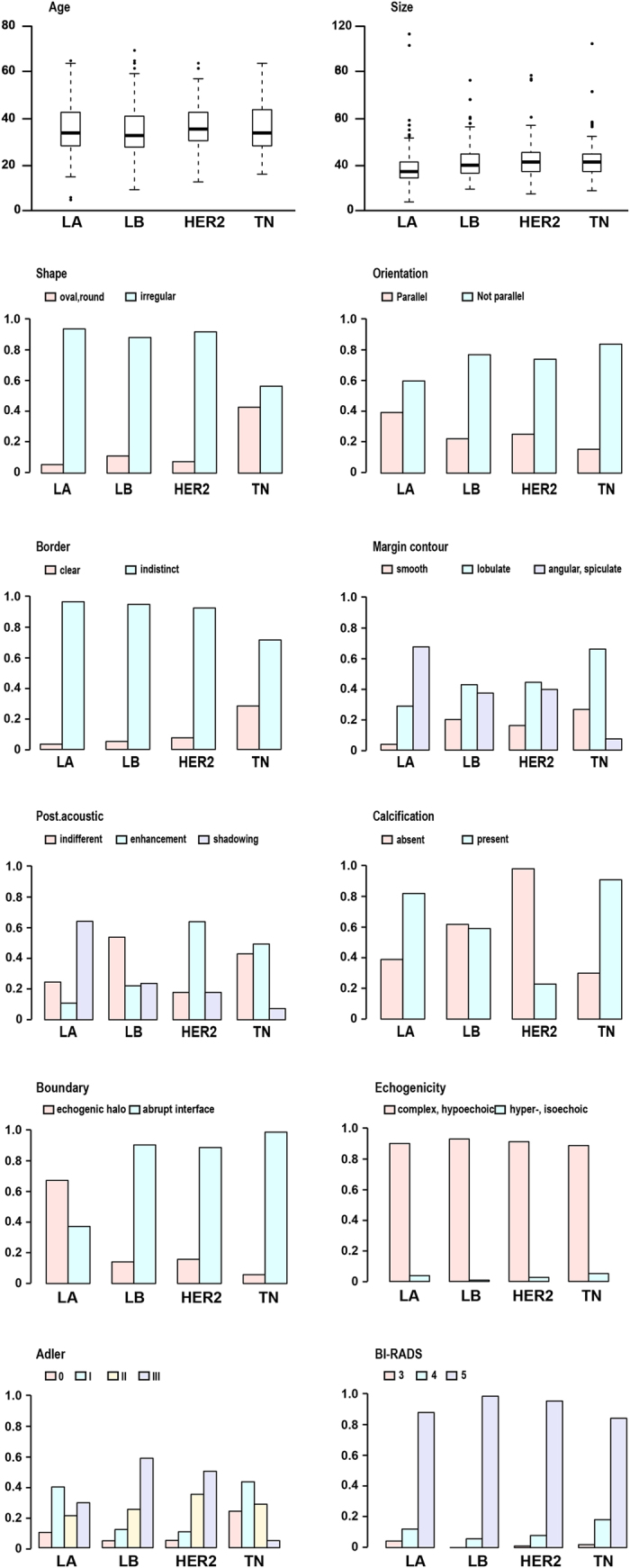
The distributions of 12 the ultrasound features of the four breast cancer subtypes (age, size, shape, orientation, border, margin contour, post-acoustic, calcification, boundary, echogenicity, Adler and BI-RADS). The colours of the columns represent the different levels of ultrasound features. HER2 patients were generally older than patients of the other three subtypes. The majority of TN tumours had oval-shaped and lobulate margins. The post-acoustic of LA tumours showed shadowing, and this was enhanced in HER2 tumors. Calcification occurred most frequently in the HER2 subtype and least frequently in the TN patients. The echogenic halo boundary occurred most frequently in the LA subtype and least frequently in the LB subtype. The Adler values of the LB and HER2 subtypes were primarily II or III, and primarily 0 or I in the TN subtype.

When analysing the four breast cancer subtypes, we found that the rates of LA, LB, HER2, and TN were 37.8%, 36.8%, 12.5% and 12.9%, respectively, as depicted in Fig. 1a. The images in Fig. 1b–e are ultrasound pictures of the four molecular subtypes, which were intuitively diverse. The appearance of each ultrasound feature in each subtype is summarized in Fig. 2.

### The ensemble decision models of breast cancer subtypes

We constructed the models using the ensemble decision approach. We randomly selected 80% of the data from each sample category (LA, LB, HER2 and TN) to construct the training set, corresponding to 256, 249, 84 and 87 patients, for a total of 676 patients. The remaining 20% of the data were used as the test set, which comprised 170 patients.

### Identifying LA breast cancer based on ultrasound features

**Figure 3:**
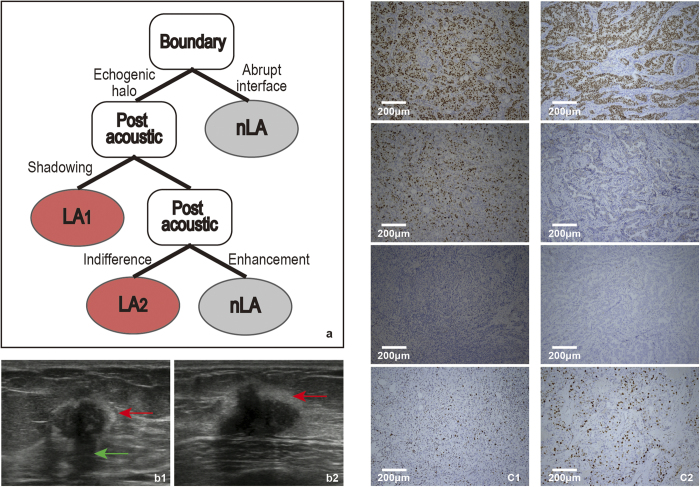
a : The model for the LA subtypes following these rules: First, the boundary was judged. If the boundary had an echogenic halo, then we judged post-acoustic. If the post-acoustic was shadowing or indifferent, the tumour was classified as the LA subtype. **b**: b1 and b2 show the ultrasound images of LA1 and LA2, respectively. The boundary of the red arrow indicates the echogenic halo, and the post-acoustic of the green arrow indicates shadowing. **c**: c1 and c2 are the IHC results of patients b1 and b2, respectively (c1, ×100; c2, ×100). From top to bottom, the three images for c1 represent ER (+), PR (+), HER2 (−), and Ki67 < 14%, and the three images for c2 represent ER (+), PR (−), HER2 (−), Ki67 < 14%. Tumour cells that stained dark brown are positive (as ER for c_1_ and c_2_). In contrast, unstained tumour cells are negative (as HER2 for c_1_ and c_2_).

We randomly selected 80% of LA data and 80% nLA data (containing the LB, HER2 and TN categories) for a total of 541 patients from the training set and we used these data to construct a decision tree. Then, we repeated this step 1,000 times, and built 1000 decision trees. We extracted the features from each decision tree and calculated their frequencies (*FV*) across the 1000 decision trees (Table 1). For each feature, we obtained the empirical null distribution by randomly permuting the category labels of the patients and determining the corresponding cutoff 
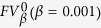
 (Table 1). Then, we selected the features (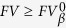, one-tailed), including boundary, post acoustic and Adler. To get the stable features, we repeated the above steps 1,000 times. The frequencies of the three features were 999, 999 and 117, respectively. We finally chose the stable features with high frequency, including boundary and post acoustic, to construct the decision model (Fig. 3a).

In the training set, the majority of patients exhibited the echogenic halo (63.7%, n = 163/256) and shadowing/indifference in terms of post-acoustic (65.6% + 25.8%, 234/256), which demonstrated that shadowing was the most common post acoustic feature, followed by indifference.

IHC was performed in the 170 patients from the test set to evaluate our model. A total of 64 patients with Estrogen Receptor (ER) and/or Progesterone Receptor (PR) positive, HER2 negative and Ki-67 < 14% were classified as LA subtype. Of the 170 patients, 57 had boundary with echogenic halo, and of these, 43 cases were of the LA subtype. In particular, there were 31 patients showing echogenic halo and post acoustic shadowing, of which 26 were the LA subtype. Additionally, 13 patients exhibited echogenic halo and post acoustic indifference, of which 8 were the LA subtype. The diagnosis of two patients whose ultrasounds featured echogenic halo (Fig. 3b1) and post-acoustic shadow (Fig. 3b1) or post-acoustic indifference (Fig. 3b2) were confirmed by the IHC results (Fig. 3c1) classified as LA subtype. Remarkably, our model yielded with an accuracy of 83.8%, sensitivity of 77.3% and specificity of 87.5%.

These data together suggested that echogenic halo was a significant feature of LA subtype. Combined echogenic halo and post-acoustic shadowing/indifference were important for distinguishing the LA subtype.

### Identifying LB breast cancer based on ultrasound features

**Figure 4:**
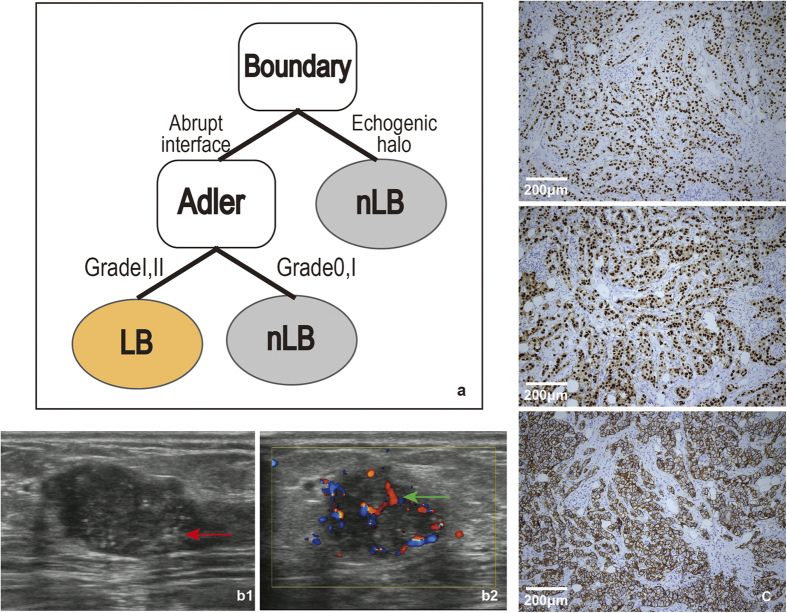
a : The model for LB subtypes followed these rules: First, the tumour boundary was judged. If the boundary did not exhibit an echogenic halo, then the Adler value was judged. If the Adler was II or III, the tumour was classified as the LB subtype. **b**: b1 and b2show the 2-dimensional and colour Doppler ultrasound images from one patient with the LB subtype. The boundary of the red arrow indicates the abrupt interface. The blue-red region of the green arrow indicates a blood vessel graded using the Adler degree. **c**: The IHC results from the patient (c, ×100). From top to bottom, the three images depict ER (+), PR (+) and HER2 (+). The tumour cells stained dark brown are positive (such as ER, PR and HER2 for c).

Likewise, we identified the ultrasound features of LB breast cancer, including boundary and Adler. The details of *FV*, cutoff and frequencies were shown in Table 1. Based on the stable features, we constructed the decision model (Fig. 4a).

In contrast to LA breast cancer, the majority of LB patients did not display echogenic halo (86.7%, n = 215/248). The Adler degree of 205 LB patients was II or III (82.7%, 205/248), while 43 showed 0 and I (17.3%, 43/248), suggesting that vascularity could be used to characterized the LB subtype.

Next, we used IHC to further validate our model. A total of 63 patients having ER or/and PR positive, HER2 negative and Ki-67 ≥ 14% or ER or/and PR positive and HER2 overexpressed or/and amplified tumor cells were classified as the LB subtype. Of the 170 patients, 114 showed boundaries without echogenic halo, of these 53 cases were of LB subtype. In particular, there were 79 patients lacked echogenic halo and vascularity, of these 60 were of LB subtype. The diagnosis of a tumour who had ultrasound features without echogenic halo (Fig. 4b1) and vascularity (Fig. 4b2) was confirmed by the IHC examination (Fig. 4c) and classified as the LB subtype. Our model yielded an accuracy of 77.4%, sensitivity of 75.9% and specificity of 78.1%.

Our results showed that the combination of echogenic halo and vascularity could help to distinguish the LB subtype, while it was necessary to identify other factors that might improve the accuracy of this model.

### Identifying HER2-amplified breast cancer based on ultrasound features

**Figure 5:**
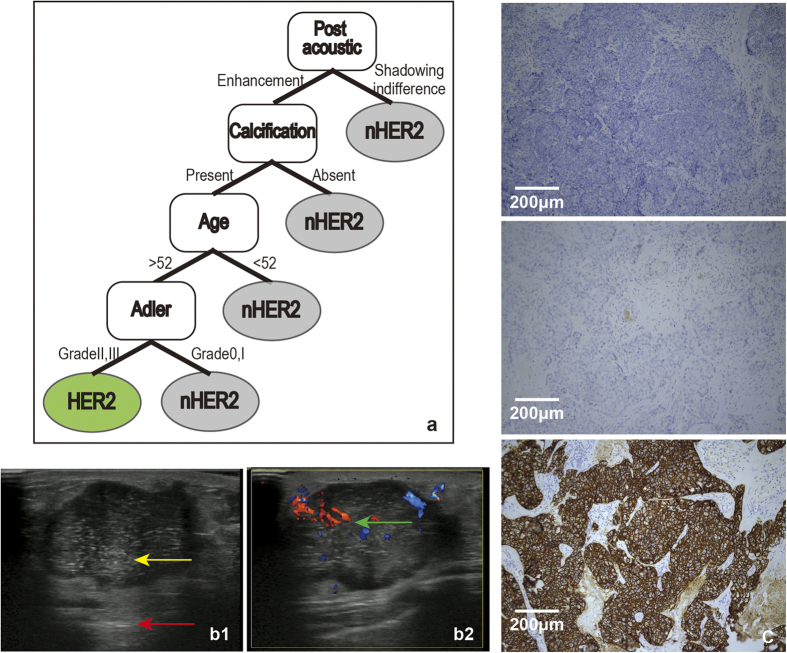
a : The model for the HER2 subtype followed these rules: First, the post-acoustic was judged. If it was enhanced, then calcification was judged. If calcification was present, then the age was judged. If the patient was older than 52, then the Adler was judged. If the Adler was II or III, the tumour was classified as being of HER2 subtype. **b**: b1 and b2 show the 2-dimensional and colour Doppler ultrasound images of one HER2 subtype patient. The image shows a red arrow that indicates post-acoustic enhancement. The punctuated, extensively hyperechoic focus is shown by the yellow arrow and indicates calcification. The blue-red region of the green arrow indicates a blood vessel graded using the Adler degree. **c**: The IHC results from the patient (c, ×100). From top to bottom, the three images for c represent ER (−), PR (−), and HER2 (+). Tumour cells stained dark brown are positive (as for HER2 in c). In contrast, the unstained tumor cells are negative (as for ER and PR in c).

Using similar approach, we chose the stable high selected features, age, post acoustic, calcification and Adler, to construct the decision model (Fig. 5a). The *FV*, cutoff and frequencies were displayed in Table 1.

The training set contained 84 HER2 subtype patients, of these 52 patients (61.9%) showed post acoustic enhancement, 66 displayed calcification (78.6%), and 49 patients were older than 52 (58.3%). In addition, the Adler degree of 70 patients was II or III (82.7%).

There were 22 patients with IHC features of HER2 subtype include ER and PR negative and HER2 overexpressed or/and amplified tumour cells. Of the 170 patients, 9 patients showed post acoustic enhancement, calcification, vascularity (Adler II or III) and were older than 52, of which 8 were of the HER2 subtype. The diagnosis of a patient whose post-acoustic showed enhancement (Fig. 5b1), exhibited calcification (Fig. 5b1), was older than 52 and abundant vascularity (Fig. 5b2) was confirmed by IHC examination (Fig. 5c) and classified as being of the HER2 subtype. The accuracy of the model was evaluated by the IHC results, showing an accuracy of 87.9%, sensitivity of 20% and specificity of 97.2%.

These data suggested that the combination of post acoustic enhancement, calcification, age older than 52 and vascularity shows high specificity, but low sensitivity, for distinguishing the HER2 subtype.

### Identifying triple-negative breast cancer based on ultrasound features

**Figure 6:**
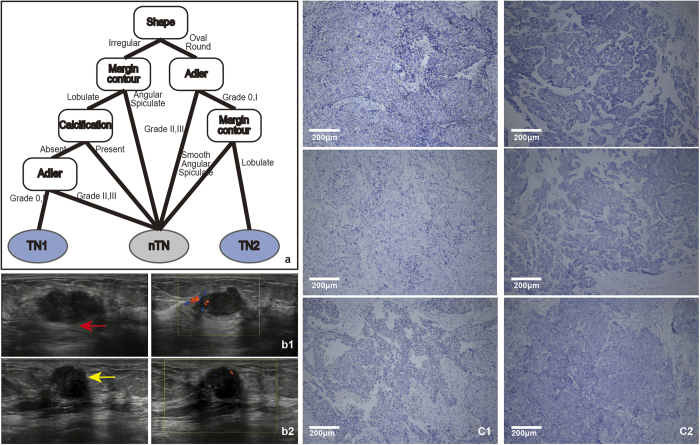
a : The model for the TN subtype followed two rules. First, the tumour shape was judged. If the shape was irregular, then the margin contour was assessed. If it was lobulate, then the calcification was judged. If calcification was absent, then the Adler degree was judged. If Adler degree was 0 or I, the tumor was classified as the TN subtype. Second, if the tumour was oval or round, then the Adler degree was judged; if the Adler was 0 or I, then the margin contour. If it was lobulate, the tumor was classified as the TN subtype. **b**: b1 and b2 show the ultrasound images of TN1 and TN2. From left to right, the two images show 2-dimensional and colour Doppler, respectively. The margin contour of red arrow depicts the lobulate margin of TN1. The margin contour of the yellow arrow depicts the micro-lobulate margin of TN2. **c**: c1 and c2 show the IHC results for the TN1 and TN2 patient (c1, ×100; c2, ×100). From top to bottom, the three images of c1 and c2 represent ER (−), PR (−), HER2 (−). Unstained tumour cells are negative (as for ER, PR and HER2 in c).

For triple-negative breast cancer, we also chose the stable high selected features of shape, margin contour, Adler and calcification (Table 1), to construct the corresponding decision model (Fig. 6a).

In the training set of 87 TN patients, 50 exhibited an irregular shape (57.5%), while 37 had an oval shape (42.5%). A total of 25 patients showed a smooth margin contour (28.7%), 56 patients had a lobulate margin contour (64.4%) and 6 patients had angular or spiculate margin contours (6.9%). Additionally, 23 patients had calcification (26.4%). 57 patients (65.5%) showed the Adler degree 0 or I. The TN subtype was characterized by both an irregular or an oval shape and smooth or lobulate margin contours. However, TN subtype patients were hypovascularity and lack of calcification.

Using IHC, TN subtype was characterized by ER, PR, and HER2 negative tumor cells, and a total of 22 patients were determined. As seen from the model, there were two categories of TN breast cancer. There were 14 patients exhibiting an irregular shape and a lobulate margin contour lacking calcification that were hypovascular, of these 10 had the TN subtype. Additionally, there were 4 patients had an oval shape, were hypovascular and showed lobulate margin contour, of which all were the TN subtype. Furthermore, the lobulate margin contour was primarily a micro-lobulate margin contour. The diagnosis of two patients, one of whom was type 1tumour with an irregular shape and a lobulate margin contour, no calcification and hypovascularity (Fig. 6b1) and another that was type 2 with an oval shape, lobulate margin contour and hypovascularity (Fig. 6b2), was confirmed by IHC examination (Fig. 6c1) as the TN subtype. The accuracy of the model was evaluated based on the results from the IHC, yielding a significant accuracy of 92.7%, a sensitivity of 63.2% and a high specificity of 98.1%.

Together, the combination of irregular shape, lobulate margin contour, lack of calcification and hypovascularity or the combination of oval shape, hypovascularity and lobulate margin contour has high accuracy and specificity but low sensitivity for distinguishing the TN subtype.

## Discussion

One challenge of breast cancer ultrasound research studies has been the development of a reliable decision-making rule for classifying patients into molecular subtypes. Accordingly, we have proposed a new method called the ensemble decision approach. From our analysis, we obtained relatively meaningful results using the ensemble decision approach. The ensemble decision approach not only identified the unique features of each molecular subtype but also generated models for distinguishing the molecular subtypes. The accuracy of the models test was high. The results described above show that a single feature could not identify the molecular subtype, but instead that an ordination of the features was valuable for molecular subtype diagnosis.

Echogenic halo and post-acoustic shadowing characterized the LA subtype. Previous studies had examined the relationship between the ultrasound features and pathological characteristics of breast cancer. It had been suggested that tumours with acoustic shadowing might be formed by desmoplastic reactions that were more likely to exist in low-grade tumours and were caused by excessive sound reflection or attenuation by the tumour compared to the surrounding tissue. It had been confirmed that tumours of the LA subtype were mostly low-grade tumors^14^. Therefore, the LA subtype was mostly^9^ associated with a post-acoustic shadowing. The echogenic halo corresponded to the histopathological features of tumour cells invading fat tissue admixed with adipocytes and elastic fibers^15^. The echogenic halo was thought to exist in low-grade and spiculate tumours^8^. Because the LA subtype was mostly of low-grade^14^ and ER (+) breast cancer accounts for the majority of cells that exhibit an echogenic halo^9^, most LA cancers also exhibited an echogenic halo. Our findings were similar to the results of Eun *et al.*, who indicated that ER-positive/PR-negative/HER2-negative breast cancers more frequently exhibited echogenic halo than triple-negative and HER2 subtype^10^.

The absence of an echogenic halo and the presence of vascularity were the characteristics of the LB subtype. The LB subtype was associated with an increased risk of relapse and most are of high-grade^16^, so that was a lack of echogenic halo. Previous studies had shown that overexpression of HER2 was closely associated with increased angiogenesis and the expression of vascular endothelial growth factor (VEGF), which mediated endothelial cell signaling and other functions^17^. There was preclinical evidence supporting the role of angiogenesis in mediating downstream HER2 signaling^18^. The results of this study showed that Luminal-B subtype tumours overexpressing HER2 demonstrate vascularity in ultrasound images.

Post-acoustic enhancement, calcification, older age and vascularity were the characteristics of the HER2 subtype. In contrast to the Luminal-A subtype, tumours with acoustic enhancement were found to be more cellular and tended to be high-grade tumours^19^ because of the reduced attenuation of the ultrasound waves compared to the surrounding tissue. The HER2 subtype was found to mainly include high-grade tumours with adverse prognoses^20^. Thus, the HER2 subtype showed post-acoustic enhancement. Our results were similar to previous findings^11^. Studies by Sung *et al.* showed that the expression of the HER2 oncogene was strongly correlated with the presence of calcification upon ultrasound^21^,^22^, and this result was similar to results obtained by many others as well as this study^23^. The HER2 oncogene was overexpressed in the HER2 subtype, which might account for the frequent observation of calcification. We found that an older age was characteristic of HER2, which was in contrast to results from other groups^16^ and this might be due to the sequential decision. In accordance with previous studies, the HER2 subtype was found to be vascular in ultrasound images.

Triple-negative breast cancer was associated with aggressive histological features, unresponsiveness to the usual endocrine treatment, a poor prognosis and a shorter survival time^24^,^25^,^26^. However, because the TN breast cancer mass could look benign, being able to discriminate TN breast cancer was of critical importance. Ko *et al.* suggested that TN breast cancers have more circumscribed margins, were hypoechoic and exhibit less calcification or posterior shadowing^10^. Dogan *et al.* found that TN breast cancers were masses without calcification, and 32% had circumscribed margins^27^. By ultrasound, Wang *et al.* concluded that TN-negative breast cancers (n = 20) were more likely to lack calcification and were more likely to present as hypoechoic (80%) masses with an irregular (54%) or lobulated (20%) shape and with distinct (40%), microlobulate (33%), smooth or circumscribed (27%) margins^5^. Ko *et al.* found that TN breast cancers were likely to be irregular (83%) or oval shaped (16%), with circumscribe (57%), angular (16%), indistinct (12%), microlobulated (9%), or spiculated (5%) margins^10^. In general, our results showed that there were two categories of imaging features for TN breast cancer. One was an irregular shape with lobulate margins, while the other was an oval shape with micro-lobulate margins. However, both categories lack vascularity, which is similar to the observations of the above study. Wojcinski *et al.* described this smooth appearance as a pushing border that was associated with a non-infiltrative process caused by rapid tumour growth^28^.

The decision models had significant applications in clinical diagnostic. For an unknown breast mass, we extracted 12 ultrasound features (Table 2), and input them into the four decision models containing 18 rules (Supplemental Information). Through the predictions of models, we obtained the categories of mass (that is, the prediction results from four decision models) and then identified the subtype. For example, assume the twelve ultrasound features of a patient were: age, 48 years; size, 23 cm; shape, irregular; orientation, parallel; margin border, indistinct; margin contour, angular/spiculate; post-acoustic, shadowing; calcification, absent; boundary, echogenic halo; echogenicity, hyper-, isoechoic; Adler, I; BI-RADS, V. Then, using the four decision models, the prediction would be “YES” only for LA subtype model and “NO” for the other three models (e.g., LB, HER2 and TN). Thus, the patient would be judged as LA subtype (for details see the Supplemental Information).

In conclusion, it is a valuable to use the ensemble decision approach to identify ultrasound and clinical features of breast cancer molecular subtypes. Distinguishing molecular subtypes using ultrasound feature-based classification models is an improvement on ultrasound diagnosis, which can serve as an effective method of auxiliary diagnosis and guide treatment in the clinical setting.

## Materials and Methods

The study protocol was approved by the Ethics Committee of Harbin Medical University (2008–0022) and written informed consent was obtained from all participants involved in the study. The methods used in this study were performed in accordance with approved guidelines. Our study sample consisted of 1,000 consecutive patients with breast cancer who underwent surgery and biopsy in the Second Affiliated Hospital of Harbin Medical University between Jan. 22, 2009 and Jan. 20, 2014, who were initially diagnosed by breast ultrasound. Patients who were treated with neo-adjuvant therapy, failed to undergo histological examination, or had multiple breast cancers were excluded. A total of 864 women (mean age 46.31 ± 9.79 years; range 11–67 years) with definite histological results were evaluated in this study.

### Ultrasound examination

All the real-time scanning was performed by a radiologist with 4 years of experience in breast ultrasound. The ultrasound was performed with a HITACHI Vision 900 system (Hitachi Medical System, Tokyo, Japan) equipped with a linear probe of 6–13 MHz. The static images and cine clips from B-mode and Doppler ultrasound were saved in the database for double-blind analysis. Three breast radiologists with 7, 9 and 13 years of clinical experience, respectively, retrospectively and independently reviewed the ultrasound images. A consensus interpretation was reached in cases of disagreement. A consensus interpretation was reached in cases of disagreement. The examined ultrasound criteria were listed, illustrated and defined in Table 2. Specifically, the Adler degree was the blood flow level of the vascularity characterization^29^. The BI-RADS was the assessment category of the breast tumour based on the Breast Imaging Reporting and Data System (BI-RADS), a standardized lexicon for ultrasound features developed in 2003 by the American College of Radiology (ACR)^30^.

### Histological examination

The experiments followed the reporting recommendations for tumour marker prognostic studies (REMARK)^31^. All the tumours were excised and stained with hematoxylin-eosin (HE). The tissues were formalin-fixed, paraffin-embedded and subsequently used for immunochemistry (IHC) staining with appropriate antibodies. The cutoff point for ER-positive, PR-positive expression was 10%. HER-2 status was graded as 0, 1+, 2+ and 3+. Only a HER-2 status of 3+ was deemed to be positive, while statuses of 0 and 1+ were deemed to be negative. Fluorescence *in situ* hybridization (FISH) was performed on all grade 2 samples. Samples with a <2-fold-change in expression were regarded as negative, and samples with a >2-fold increase were regarded as positive for gene amplification^24^,^32^. Ki67 was visually scored for the percentage of tumour cell nuclei with positive immunostaining above background. Over 14% was considered high expression, and less than 14% was considered low expression^33^.

### Molecular subtypes of breast cancer

Breast cancer molecular subtypes were categorized according to the immunohistochemistry results for ER, PR, HER2 and Ki-67, as recommended by the 12^th^ International Breast Conference^30^, as follows:

Luminal A type (LA): ER or/and PR positive, HER2 negative and Ki-67 < 14%;

Luminal B type (LB): ER or/and PR positive, HER2 negative and Ki-67 ≥ 14%, ER or/and PR positive and HER2 overexpressed or/and amplified;

HER2 amplified type (HER2): ER and PR negative and HER2 overexpressed or/and amplified;

Triple-Negative type (TN): ER, PR and HER2 negative.

### Ensemble decision approach

We proposed an ensemble decision approach based on a recursive partition tree, using the following basic procedures. First, a resampling technique was used to construct a training set and a test set for learning and testing, respectively. Second, a binary tree was grown on the outset, with 80% of the data randomly selected from each sample category of the training set by a recursive partition algorithm. This step was repeated 1,000 times, so that 1,000 trees and the FV of each feature were obtained. Third, feature selection was optimized using a method we developed.

#### Construction of a features matrix

The ultrasound matrix could be represented by an m*n matrix, in which m represented the patients and n represented the feature. In the matrix, each element X = (x_pq_) represented the *q*th ultrasound features of the *p*th sample (X_p_). Each sample could be described by a feature vector X_i_ = (x_i1_,…,x_iq_). The samples were divided into four classes, LA, LB, HER2 and TN^3^.

#### Ensemble feature selection

The proposed ensemble selection was a data-mining method based on decision trees, which had previously been applied as an effective solution to classification and prediction problems^12^. The meaning of the decision tree was a sequence of binary splits of the data, separating one class from the other classes as effectively as possible^34^. The recursive partition tree is one of the most effective methods used for constructing decision trees^35^.

#### Construction of the training set and the test set

The given data were divided into two sets. The training set was used to build the classification model, and the test data set was used to validate the model. First, we randomly selected 80% of the data from each sample category to construct the training set, and the remaining 20% of the data were used as the test set. Then, from the training set, we randomly selected 80% of the data from one type and 80% of the data from the remaining types, called the outer set, to construct the decision tree (the following description used HER2 as an example).

#### Algorithm of the recursive partition tree

The trees were structured as a root, internal and leaf nodes. A binary tree was grown from the outer set using a recursive partition algorithm. Depending on whether a particular selected predictor was above a chosen cutoff value, the samples were divided into smaller and smaller groups. If the ultrasound feature leads to minimal impurity at the node, this feature was selected at the node of the tree. At each internal node, a decision was made with regard to the choice of a feature and a threshold value (cut-off), such that class impurity was reduced to a minimum when a branch was created by an the induction rule^13^.

#### Selection of relevant ultrasound features

When tree growth was stopped, we extracted the ultrasound features at the nodes. A subset of the ultrasound features was obtained from the particular outer set, denoted {*F*_1_, *F*_2_,…, *Fq*} We defined *FV* as the magnitude of the relevance intensity, which could be used to calculate whether an ultrasound feature was relevant to one category as follows:


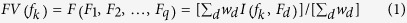


*f*_k_ denoted for a particular ultrasound feature; *w*_d_ was the weight, a measure of the classification performance of *F*_d_; 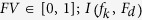 was an indicator functions:


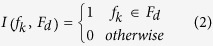


To build the distribution of the *FV*, we randomly assigned a category label to each patient in a process, called the permutation approach. *FV*(*f*_*k*_) was computed using the permutation results. Then, *FV*^0^(*f*_*k*_), the empirical null distribution, was obtained. The critical value, 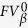, was obtained based on the given empirical *FV*^0^(*f*_*k*_) and a specified significance level, *β*(*e.g*. 0.001). If 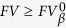 (one-tailed), the ultrasound feature was chosen. We repeated this step 1,000 times and selected the stable highly frequent ultrasound features from an ultrasound features subset. The final decision tree was constructed from the feature group.

#### Evaluation of the ensemble decision model

We used the *χ*^2^ statistic method to assess the accuracy of the extracted ultrasound features subset,


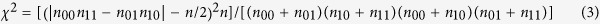


Where, *n* = *n*_00_ + *n*_01_ + *n*_10_ + *n*_11_, *n*_00_ was the frequency of true negatives, *n*_01_ was the frequency of false positives, *n*_10_ was the frequency of false negatives and *n*_11_ was the frequency of true positives. This statistic followed an asymptotic *χ*^2^ distribution with one degree of freedom.

**Figure 7:**
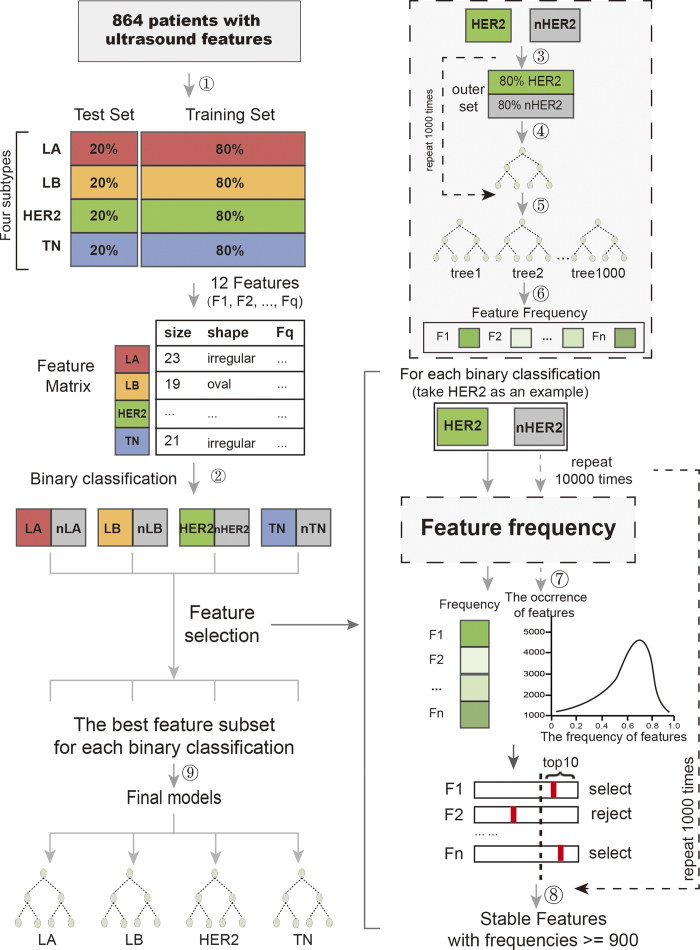
The step-by-step protocol for the ensemble approach computational algorithm. ① Construct the training and test set by sampling patients. The training set and test set contain 80% and 20% of the initial patients for each subtype, respectively. ② Binary classification: LA, LB and TN are classified as one category, called nHER2 (take HER2 as an example). ③ Randomly sample patients, 80% HER2 and 80% nHER2, from the training set, called the outer set. ④ Grow a tree on the particular set obtained from step 2. ⑤ After repeating steps 2-4 1,000 times, we obtained 1,000 trees. ⑥ Count the occurrence frequencies of all features in the above 1,000 trees. ⑦ Randomly assign a label to each patient. Then, use the shuffled data to conduct steps 1-5. Compute the relevance intensity, (*FV*) 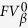, for each feature. Repeat the permutation 10,000 times to obtain an empirical threshold for the specified significance level of *β*. ⑧ After repeating steps 2–6 1,000 times, we obtained the stable high occurrence frequency of features. ⑨ Construct the model based on the selected features.

The computational process was implemented on the R platform. The specific workflow was shown in Fig. 7.

## Additional Information

**How to cite this article**: Zhang, L. *et al.* Identifying ultrasound and clinical features of breast cancer molecular subtypes by ensemble decision. *Sci. Rep.* **5**, 11085; doi: 10.1038/srep11085 (2015).

## Supplementary Material

Supplementary Information

## Figures and Tables

**Table 1:** **The FV, cutoff and frequencies for each subtype.**

**Features**	**LA**				**LB**				**HER2**				**TN**			
	***FV***	**Cutoff**	**Frequencies**	**Final selection**	***FV***	**Cutoff**	**Frequencies**	**Final selection**	***FV***	**Cutoff**	**Frequencies**	**Final selection**	***FV***	**Cutoff**	**Frequencies**	**Final selection**
Age	0.792	1.000	0	no	0.978	1.000	0	no	0.972	0.779	998	yes	0.551	0.771	0	no
Size	0.722	1.000	0	no	0.806	1.000	0	no	0.756	0.851	0	no	0.964	0.774	885	no
Shape	0	0.956	0	no	0.034	0.952	0	no	0.011	0.428	0	no	0.999	0.463	992	yes
Orientation	0.002	0.962	0	no	0.218	0.972	0	no	0.183	0.451	0	no	0.013	0.452	0	no
Margin border	0	0.945	0	no	0.252	0.956	0	no	0	0.539	0	no	0.036	0.399	996	yes
Margin contour	0.566	0.983	0	no	0.741	0.985	0	no	0.117	0.569	0	no	0.845	0.452	462	no
Post. acoustic	1	0.987	999	yes	0.992	0.987	397	no	1	0.532	999	yes	0.545	0.496	662	no
Calcification	0.245	0.966	0	no	0.608	0.966	0	no	1	0.437	999	yes	0.825	0.421	993	yes
Boundary	1	0.926	999	yes	0.999	0.954	999	yes	0.026	0.402	0	no	0.461	0.432	0	no
Echogenicity	0	0.913	0	no	0	0.937	0	no	0	0.100	0	no	0	0.117	0	no
Adler	1	0.988	117	no	1	0.993	999	yes	0.567	0.503	934	yes	1	0.662	999	yes
BI-RADS	0.363	0.956	0	no	0.824	0.966	0	no	0	0.441	0	no	0.209	0.428	0	no

**Table 2:** **Definition of the ultrasound features criteria.**

**Variables**		**Definition**
Size		Maximum diameter of the tumor by ultrasound
Shape	Oval, round	Oval, spherical or round
	irregular	Not round or oval
Orientation	Parallel	Long axis of lesion parallels the skin line
	Not parallel	Long axis, not oriented along the skin line
Margin border	circumscribed	A margin that is well defined or sharp, with an abrupt transition
	indistinct	No clear demarcation between mass and its surrounding tissue
Margin contour	smooth	Smooth, even margin without any irregularity
	lobulate	Short cycle undulations impart a scalloped appearance to the margin of the mass
	Angular, spiculate	Margin is formed or characterized by sharp lines projecting from the mass
Post. acoustic	Indifferent	No shadowing or enhancement
	Enhancement	Increased posterior echo
	Shadowing	Decreased posterior echo and combined
Calcification	Absent	No punctuated extensively hyper-echoic foci
	Present	Punctuated extensively hyper-echoic foci
Boundary	Abrupt interface	No thin capsule or echoic halo
	Echogenic Halo	Blurred, irregular hyperechoic rim around the lesion
Echogenicity	Hyper-, isoechoic	Hyper- or isoechogenicity compared to fat, e.g., fibroglandular tissue
	Complex, hypoechoic	Hypoechoic compared to fat tissue
Adler	0	Vascularity not present
	I	1-2 spot vessels, caliber shorter than 1 mm
	II	1-2 vessels, longer than the radius of the tumor
	III	More than 4 vessels
BI-RADS	I	No lesion found
	II	Benign finding
	III	Probably benign finding
	IV	Suspicious abnormality
	V	Highly suggestive of malignancy
