# Supplementary material for: Identifying ultrasound and clinical features of breast cancer molecular subtypes by ensemble decision
Source: Sci Rep. 2015 Jun 5;5:11085. doi: 10.1038/srep11085 (PMC4457139; doi:10.1038/srep11085)
Supplement: Supplementary Information [file srep11085-s1.doc]

**Supplementary Information**

**Identifying ultrasound and clinical features of breast cancer molecular subtypes by ensemble decision**

Lei Zhang^1^, Jing Li^2^, Yun Xiao^3^, Hao Cui^1^, Guoqing Du^1^, Ying Wang^4^, Ziyao Li^1^, Tong Wu^1^, Xia Li^3*^, Jiawei Tian^1*^

^1^Department of Ultrasound, The Second Affiliated Hospital of Harbin Medical University, Harbin, Heilongjiang ,China.

^2^Department of Ultrasonic medicine, The 1st Affiliated Hospital of Heilongjiang University of Chinese Medicine, Harbin, Heilongjiang, China.

^3^ College of Bioinformatics Science and Technology, Harbin Medical University,
Harbin, Heilongjiang, China.

^4^Department of general surgery, The Second Hospital of Hebei Medical Universtiy, Shijiazhuang, Hebei, China

Correspondence: Jiawei Tian, The Second Affiliated Hospital of Harbin Medical University, Harbin, Heilongjiang, China,150086. Tel：+86(0451)86605811;  Fax：+86(0451)86675845; E-mail: [jwtian2004@163.com](mailto:jwtian2004@163.com). Xia Li, College of Bioinformatics Science and Technology, Harbin Medical University, Harbin, Heilongjiang, China, 150081. Tel：+86(0451)86615922; E-mail: [lixia@hrbmu.edu.cn](mailto:lixia@hrbmu.edu.cn).

^*^These correspondence authors contributed equally to this work.

**The decision rules from the four models**

The 4 decision rules from LA subtype model


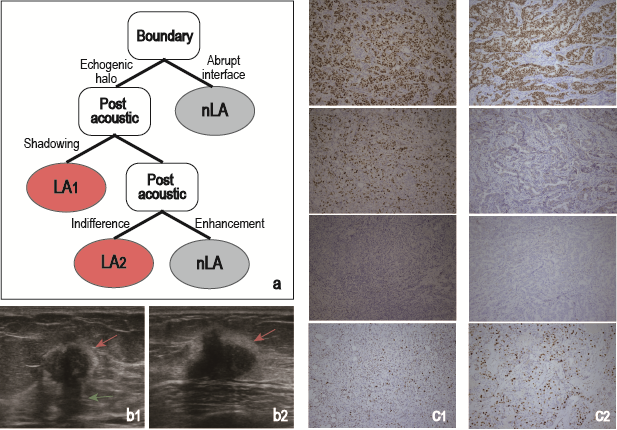


Supplementary Figure S1: The decision model of LA subtype

1.

2.

3.

4.

The 3 decision rules of LB subtype model


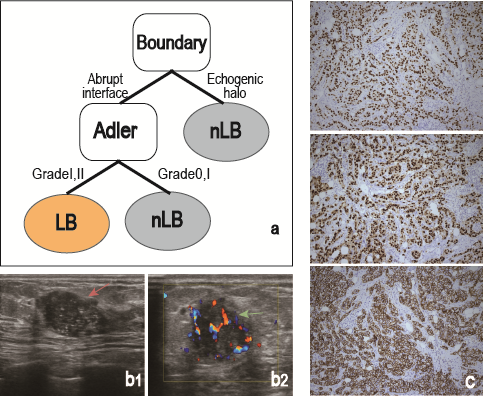


Supplementary Figure S2: The decision model of LB subtype

5.

6.

7.

The 5 decision rules of HER2 subtype model


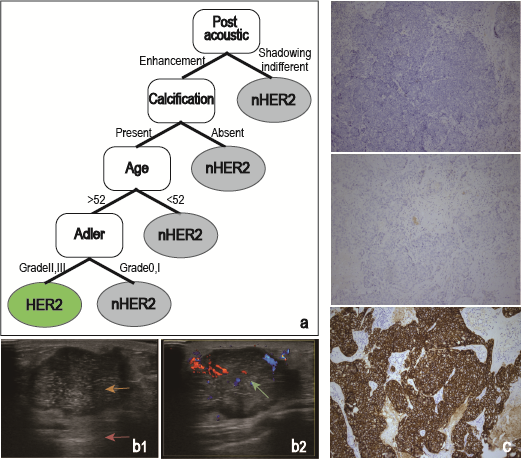


Supplementary Figure S3: The decision model of HER2 subtype

8.

9.

10.

11.

12.

The 6 decision rules of TN subtype model


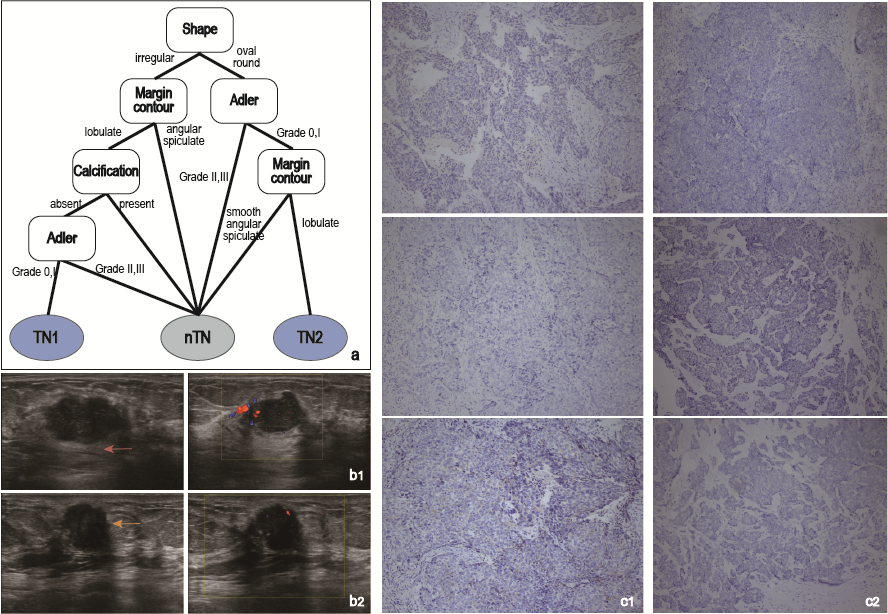


Supplementary Figure S4: The decision model of TN subtype

13.

14.

15.

16.

17.

18.

As an example, consider a patient with the following 12 ultrasound features: age, 48 years; size, 23 cm; shape, irregular; orientation, parallel; margin border, indistinct; margin contour, angular/spiculate; post-acoustic, shadowing; calcification, absent; boundary, echogenic halo; echogenicity, hyper-, isoechoic; Adler, I; BI-RADS, V. (Supplementary Figure S5)

We then input these 12 features into the four decision models:

1. LA subtype model: the boundary of the echogenic halo and post-acoustic shadowing was in line with No. 2 rule of the LA subtype, suggesting that this patient was of the LA subtype.
2. LB subtype model: based on the boundary of echogenic halo, rule No. 7 of the LB suggested the patient was of the nLB subtype.
3. HER2 subtype model: based on the post-acoustic shadowing, rule No. 12 of the HER2 subtype suggested the patient was of the nHER2 subtype.
4. TN subtype model: based on the irregular shape and angular/speculated margin contour, rule No. 14 for the TN subtype suggested the patient was of the nTN subtype.

In summary, the patient tumour was therefore determined to be of the LA subtype.


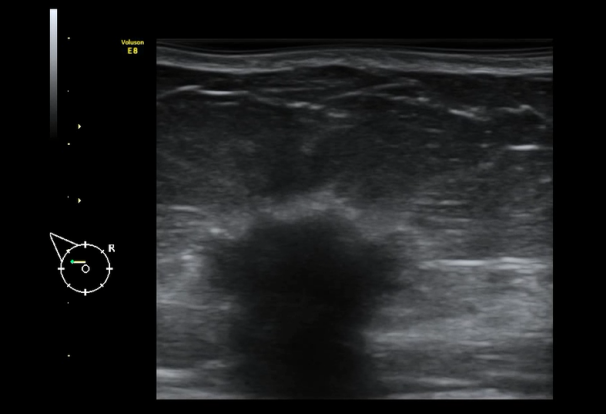

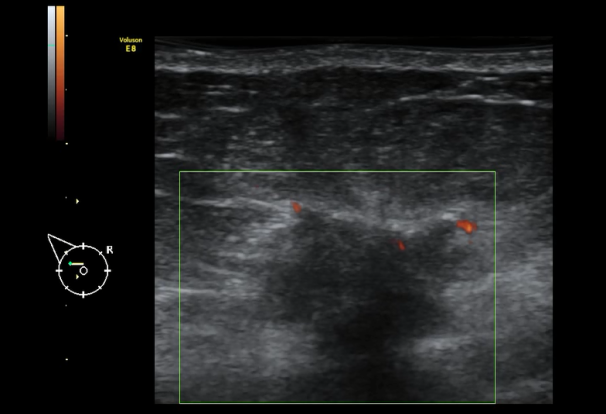


Supplementary Figure S5: The ultrasound picture of the patient.
